# Descriptors for Pentane-2,4-dione and Its Derivatives

**DOI:** 10.1007/s10953-017-0667-y

**Published:** 2017-08-19

**Authors:** Michael H. Abraham, William E. Acree

**Affiliations:** 1 Department of Chemistry, University College London, 20 Gordon St, London, WC1H 0AJ UK; 2 Department of Chemistry, University of North Texas, 1155 Union Circle Drive #305070, Denton, TX 76203-5017 USA

**Keywords:** Pentane-2,4-dione, Partition coefficients, Absolv descriptors, Acetylacetone, Linear free energy relationships

## Abstract

We have used equations for partition coefficients of compounds from water and the gas phase to various solvents to obtain descriptors for pentane-2,4-dione and 21 of its derivatives. These descriptors can then be used to estimate further partition coefficients into a wide variety of solvents. The descriptors also yield information about the properties of pentane-2,4-dione and its derivatives. Pentane-2,4-dione and its alkyl derivatives are quite polar, with substantial hydrogen bond basicity but with no hydrogen bond acidity. In contrast 1,1,1-trifluoropentane-2,4-dione and hexafluoropentan-2,4-dione have significant hydrogen bond acidities.

## Introduction

The extraction of metal ions from aqueous solution into organic phases by the use of organic ligands is of great importance in the separation and purification of metals [1]. Quantitative studies of the extraction process require a knowledge of the water–solvent partition of the organic ligand, and so any method of estimating or predicting partition coefficients of organic ligands would be a considerable help in the design of new or novel extraction systems. We have set out a systematic method for the determination of properties or ‘descriptors’ of molecules [2–5], mostly using experimental values of water to solvent partition coefficients. These descriptors, known as Abraham or Absolv descriptors can then be used to estimate partition coefficients for other water–solvent systems, as well as numerous physicochemical, environmental and biological properties. We have already used this method to determine descriptors for organophosphorus extractants [6]. Another well-known class of extracting agents is based on pentane-2,4-dione, or acetylacetone and its derivatives. Although we have preliminary descriptors for acetylacetone and some derivatives [7, 8], these were based on limited data, and so we have re-determined descriptors for acetylacetone itself, and have obtained new descriptors for 20 derivatives that have been used as extraction agents.

In solution, acetylacetone and its derivatives exist as a mixture of keto and enol forms, the proportion of which depends on the solvent. If the keto–enol equilibrium constant is known in a number of solvents for which the corresponding water–solvent partition coefficients are known, then it is possible to determine descriptors separately for the keto and enol forms [9]. For many of the compounds that we consider in this work, the keto–enol equilibrium constants are not known, and so we use the experimental water–solvent partition coefficients to obtain descriptors for the keto–enol mixture. These obtained descriptors can then be used to predict further experimental partition coefficients into a wide range of solvents.

## Methodology

We start with our well-known linear free energy relationships, LFERS, Eqs. 1 and 2 [2–5] for the partition of neutral molecules (non-electrolytes) from water to another solvent or solvent system,

1 $$ { \log }_{ 10} P = c + e\varvec{E} + {\mathbf{s}}S + \, a\varvec{A} + \, b\varvec{B} + \, v\varvec{V} $$

2 $$ { \log }_{ 10} K = c + e\varvec{E} + {\mathbf{s}}S + \, a\varvec{A} + \, b\varvec{B} + \, l\varvec{L} $$

In Eq. 1, the dependent variable is log_10_ *P*, where *P* is the water to solvent partition coefficient for a series of non-electrolytes in a given water to solvent system. In Eq. 2, the dependent variable is log_10_ *K*, where *K* is the gas phase to solvent system partition coefficient. The independent variables are descriptors as described previously [2–5]. ***E*** is the non-electrolyte (or solute) excess molar refractivity in units of (cm^3^·mol^−1^)/10, ***S*** is the solute dipolarity/polarizability, ***A*** and ***B*** are the overall or summation solute hydrogen bond acidity and basicity, ***V*** is the solute McGowan characteristic volume in units of (cm^3^·mol^−1^)/100, and ***L*** is log_10_ *K16*, where *K16* is the gas to hexadecane partition coefficient at 298 K. The use of Eqs. 1 and 2 has been reviewed [2–5]; the review of Clarke and Mallon [5] is particularly exhaustive.

In order to obtain descriptors we first need LFERs, based on Eq. 1 for partition from water to various solvent systems. The coefficients in Eq. 1 for partition from water into wet (water saturated) solvents are in Table 1 [6]. Then if we have log_10_ *P* values for a given solute in systems for which we have descriptors, we can determine values of the descriptors in Eq. 1 by solution of a set of simultaneous equations. We usually have more equations than we have unknowns (i.e. the descriptors). In this case, use of Microsoft ‘Solver’ is a very convenient way of solving the set of equations by trial-and-error. The solution is the set of descriptors that gives the best fit of the dependent variable. The solution is greatly helped if we have prior knowledge of some of the descriptors. The ***E***-descriptor can be obtained from a refractive index at 293 K (for liquid solutes), or can be calculated from an estimated refractive index [10]. Both the available software programs [7, 8] for descriptors give calculated values of ***E***. The ***V***-descriptor can easily be calculated from its molecular formula [2, 11]. Thus we have three descriptors in Eq. 1 to determine (***S***, ***A*** and ***B***). However we can convert all values of log_10_ *P* into corresponding gas–solvent partition coefficients, as log_10_ *K*, through Eq. 3 where *K*_w_ is the gas to water partition coefficient, all partition coefficients being at 298 K. Note that *K*_w_ has no units. We take log_10_ *K*_w_ as another unknown ‘descriptor’ and use both Eqs. 1 and 2 in our set of simultaneous equations. Coefficients in Eq. 2 are given in Table 1. Then even if we have a limited number of log_10_ *P* values, say only five, we then have five equations in log_10_ *P* and five equations in log_10_ *K*. We also have two equations for log_10_ *K*_w_, see Table 1, making a total of 12 equations from which to derive five unknowns (***S***, ***A***, ***B, L*** and log_10_ *K*_w_). This is the procedure we use for the determination of descriptors.

**Table 1:** Coefficients in Eqs. 1 and 2 for partition of solutes from water and the gas phase to wet organic solvents at 298 K

Solvent	*c*	*e*	*s*	*a*	*b*	*l*	*v*
Coefficients in Eq. 1							
Butan-1-ol	0.376	0.434	−0.718	−0.097	−2.350	0.000	2.682
Pentan-1-ol	0.185	0.367	−0.732	0.105	−3.100	0.000	3.395
Hexan-1-ol	−0.006	0.460	−0.940	0.142	−3.284	0.000	3.792
Heptan-1-ol	0.041	0.497	−0.976	0.030	−3.438	0.000	3.859
Octan-1-ol	0.088	0.562	−1.054	0.034	−3.460	0.000	3.814
Nonan-1-ol	−0.041	0.562	−1.103	0.090	−3.540	0.000	3.922
Decan-1-ol	−0.136	0.542	−0.989	0.046	−3.722	0.000	3.996
Isobutanol	0.249	0.480	−0.639	−0.050	−2.284	0.000	2.758
Dichloromethane	0.319	0.102	−0.187	−3.058	−4.090	0.000	4.324
Trichloromethane	0.191	0.105	−0.403	−3.112	−3.514	0.000	4.395
Tetrachloromethane	0.199	0.523	−1.159	−3.560	−4.594	0.000	4.618
1,2-Dichloroethane	0.183	0.294	−0.134	−2.801	−4.291	0.000	4.180
Hexane	0.333	0.560	−1.710	−3.578	−4.939	0.000	4.463
Heptane	0.297	0.634	−1.755	−3.571	−4.946	0.000	4.488
Octane	0.241	0.690	−1.769	−3.545	−5.011	0.000	4.511
Decane	0.172	0.726	−1.750	−3.446	−4.496	0.000	4.489
Undecane	0.058	0.603	−1.661	−3.421	−5.120	0.000	4.619
Dodecane	0.114	0.668	−1.644	−3.545	−5.006	0.000	4.459
Hexadecane	0.087	0.667	−1.617	−3.587	−4.869	0.000	4.433
Cyclohexane	0.159	0.784	−1.678	−3.740	−4.929	0.000	4.577
Methylcyclohexane	0.246	0.782	−1.982	−3.517	−4.293	0.000	4.528
Isooctane	0.318	0.555	−1.737	−3.677	−4.864	0.000	4.417
Benzene	0.142	0.464	−0.588	−3.099	−4.625	0.000	4.491
Toluene	0.143	0.527	−0.720	−3.010	−4.824	0.000	4.545
Fluorobenzene	0.139	0.152	−0.374	−3.030	−4.601	0.000	4.540
Chlorobenzene	0.065	0.381	−0.521	−3.183	−4.700	0.000	4.614
Bromobenzene	−0.017	0.436	−0.424	−3.174	−4.558	0.000	4.445
Iodobenzene	−0.192	0.298	−0.308	−3.213	−4.653	0.000	4.588
Nitrobenzene	−0.152	0.525	0.081	−2.332	−4.494	0.000	4.187
Benzonitrile	0.097	0.285	0.059	−1.605	−4.562	0.000	4.028
Diethyl ether	0.248	0.561	−1.016	−0.226	−4.553	0.000	4.075
Diisopropylether	0.472	0.413	−0.745	−0.632	−5.251	0.000	4.059
Dibutylether	0.252	0.677	−1.506	−0.807	−5.249	0.000	4.815
Ethyl acetate	0.441	0.591	−0.699	−0.325	−4.261	0.000	3.666
Butyl acetate	−0.475	0.428	−0.094	−0.241	−4.151	0.000	4.046
Methyl isobutyl ketone	0.383	0.801	−0.831	−0.121	−4.441	0.000	3.876
Tributylphosphate	0.327	0.570	−0.837	−1.069	−4.333	0.000	3.919
Gas–water	−0.994	0.577	2.549	3.813	4.841	0.000	−0.869
Coefficients in Eq. 2							
Butan-1-ol	−0.095	0.262	1.396	3.405	2.565	0.523	0.000
Pentan-1-ol	−0.107	−0.001	1.188	3.614	1.671	0.721	0.000
Hexan-1-ol	−0.302	−0.046	0.880	3.609	1.785	0.824	0.000
Heptan-1-ol	−0.159	0.018	0.825	3.539	1.425	0.830	0.000
Octan-1-ol	−0.222	0.088	0.701	3.478	1.477	0.851	0.000
Nonan-1-ol	−0.197	0.141	0.694	3.616	1.299	0.827	0.000
Decan-1-ol	−0.302	0.233	0.741	3.531	1.177	0.835	0.000
Isobutanol	0.000	0.000	0.000	0.000	0.000	0.000	0.000
Dichloromethane	0.192	−0.572	1.492	0.460	0.847	0.965	0.000
Trichloromethane	0.157	−0.560	1.259	0.374	1.333	0.976	0.000
Tetrachloromethane	0.217	−0.435	0.554	0.000	0.000	1.069	0.000
1,2-Dichloroethane	0.017	−0.337	1.600	0.774	0.637	0.921	0.000
Hexane	0.320	0.000	0.000	0.000	0.000	0.945	0.000
Heptane	0.284	0.000	0.000	0.000	0.000	0.950	0.000
Octane	0.219	0.000	0.000	0.000	0.000	0.960	0.000
Decane	0.159	0.000	0.000	0.000	0.000	0.972	0.000
Undecane	0.113	0.000	0.000	0.000	0.000	0.971	0.000
Dodecane	0.053	0.000	0.000	0.000	0.000	0.986	0.000
Hexadecane	0.000	0.000	0.000	0.000	0.000	1.000	0.000
Cyclohexane	0.163	−0.110	0.000	0.000	0.000	1.013	0.000
Methylcyclohexane	0.318	−0.215	0.000	0.000	0.000	1.012	0.000
Isooctane	0.264	−0.230	0.000	0.000	0.000	0.975	0.000
Benzene	0.107	−0.313	1.053	0.457	0.169	1.020	0.000
Toluene	0.121	−0.222	0.938	0.467	0.099	1.012	0.000
Fluorobenzene	0.181	−0.621	1.432	0.647	0.000	0.986	0.000
Chlorobenzene	0.064	−0.399	1.151	0.313	0.171	1.032	0.000
Bromobenzene	−0.064	−0.326	1.261	0.323	0.292	1.002	0.000
Iodobenzene	−0.171	−0.192	1.197	0.245	0.245	1.002	0.000
Nitrobenzene	−0.295	0.121	1.682	1.247	0.370	0.915	0.000
Benzonitrile	−0.075	−0.341	1.798	2.030	0.291	0.880	0.000
Diethylether	0.206	−0.169	0.873	3.402	0.000	0.882	0.000
Dipropylether	0.065	−0.202	0.776	3.074	0.000	0.948	0.000
Diisopropylether	0.114	−0.032	0.685	3.108	0.000	0.940	0.000
Dibutylether	0.369	−0.216	0.026	2.626	−0.499	1.124	0.000
Ethyl acetate	0.130	0.031	1.202	3.199	0.463	0.828	0.000
Butyl acetate	−0.664	0.061	1.671	3.373	0.824	0.832	0.000
Methyl isobutyl ketone	0.244	0.183	0.987	3.418	0.323	0.854	0.000
Tributylphosphate	0.097	−0.098	1.103	2.411	0.588	0.844	0.000
Gas–water	−1.271	0.822	2.743	3.904	4.814	−0.213	0.000

3 $$ { \log }_{ 10} P = { \log }_{ 10} K{-}{ \log }_{ 10} K_{\text{w}} $$

## Results

The required values of log_10_ *P* that we need to initiate the calculation of descriptors are known for acetylacetone and for a number of substituted compounds. Quite fortunately, Leo [12] has collected these log_10_ *P* values, many of which are scattered over the literature, and lists them in his software program ‘BioLoom’. The log_10_ *P* values that we use are nearly all from BioLoom. We start with acetylacetone itself. A value of ***E*** = 0.412 from an experimental value of the refractive index [13] is available, and ***V*** = 0.8445 [2, 11]. Partition coefficients into no less than 26 solvents are available [12]. Values of log_10_ *P* into hexane, decane and butyl acetate were well out of line and were not used, leaving 23 data points. We have also 23 corresponding values of log_10_ *K*, two equations in log_10_ *K*_w_ and one equation for the NIST Kovats GC retention index, GCRI, leading to a total set of 49 equations. A value of GCRI = 790 for acetylacetone is listed in ChemSpider [13]. We have used NIST Kovats GC values to obtain Eq. 4:

4 $$ {\text{GCRI }} = { 69}. 6 { } + { 12}. 1\varvec{E} + { 76}. 3\varvec{S} + { 2}00.0\varvec{L} $$

$$ N = { 286},SD = { 46}. 4,R^{2} = \, 0. 9 9 2,F = { 12}0 7 9,{{ PRESS }} = { 634316},Q^{2} = \, 0. 9 9 2,PSD = { 47}. 7 $$

Here and elsewhere, *N* is the number of data points (compounds), *SD* is the regression standard deviation, *R* is the correlation coefficient, *F* is the *F*-statistic, *PRESS* and *Q* ^2^ are the leave-one-out statistics and *PSD* is the predictive standard deviation [14]. In order not to bias the results, we use GCRI/100 in the set of simultaneous equations.

The total of 49 simultaneous equations were solved to yield the descriptors given in Table 2 with *SD* = 0.149 log_10_ units. The 23 observed and calculated values of log_10_ *P* are in Table 3. For this set of data the Absolute Error (*AE*) = 0.018 and *SD* = 0.146 log_10_ units. The descriptors for acetylacetone are not unusual, except that ***A*** = 0. It might be expected that acetylacetone would have some hydrogen bond acidity through the ‘active’ CH_2_ group but we have enough data, with 49 equations, to be reasonably certain that ***A*** = 0.

**Table 2:** Descriptors for pentane-2,4-dione and some of its derivatives

Compound	***E***	***S***	***A***	***B***	***V***	***L***	log_10_ *K*_w_
Pentane-2,4-dione	0.412	0.80	0.00	0.62	0.8445	3.347	3.59
Hexane-2,4-dione	0.381	0.78	0.00	0.65	0.9854	3.727	3.51
Heptane-2,4-dione	0.385	0.73	0.00	0.64	1.1263	4.204	3.22
Octane-2,4-dione	0.380	0.73	0.00	0.65	1.2672	4.780	3.14
Nonane-2,4-dione	0.380	0.76	0.00	0.66	1.4081	5.308	3.15
Heptane-3,5-dione	0.385	0.78	0.00	0.66	1.1263	4.217	3.45
Octane-3,5-dione	0.380	0.75	0.00	0.65	1.2672	4.787	3.19
Nonane-4,6-dione	0.399	0.77	0.00	0.66	1.4081	5.242	3.20
Undecane-5,7-dione	0.370	0.76	0.00	0.68	1.6899	6.223	3.03
Tridecane-6,8-dione	0.370	0.75	0.00	0.67	1.9717	7.371	2.70
5,5-Dimethylhexane-2,4-dione	0.380	0.81	0.00	0.73	1.2672	4.798	3.75
2,6-Dimethylheptane-3,5-dione	0.380	0.72	0.00	0.65	1.4081	5.206	2.99
2,8-Dimethylnonane-4,6-dione	0.370	0.77	0.00	0.72	1.6899	6.224	3.24
3-Methyl-2,4-pentanedione	0.380	0.77	0.00	0.65	0.9854	3.781	3.48
2,2,6,6-Tetramethylheptane-3,5-dione	0.360	0.78	0.00	0.72	1.6899	6.247	3.25
Benzoylacetone	1.000	1.06	0.00	0.60	1.3114	6.012	4.05
1,1,1-Trifluorobenzoylacetone	0.690	0.69	0.00	0.77	1.3645	5.494	3.72
Thenoylacetone	1.100	1.00	0.00	0.69	1.2361	5.810	4.46
1,1,1-Trifluoro-2-thenoylacetone	0.530	0.91	0.00	0.83	1.2892	5.210	4.58
Trifluoroacetylacetone	0.106	0.47	0.16	0.72	0.8976	2.878	3.60
Hexafluoroacetylacetone^a^	−0.217	0.07	0.32	0.80	0.9507	2.340	3.34

^a^Provisional only

**Table 3:** Calculated and observed values of log_10_ *P* for water–solvent partition of 2,4-pentanedione (acac)

Solvent	Calc	Obs
Pentan-1-ol	0.70	0.43
Hexan-1-ol	0.60	0.35
Heptan-1-ol	0.59	0.32
Octan-1-ol	0.55	0.40
Nonan-1-ol	0.43	0.26
Dichloromethane	1.33	1.33
Trichloromethane	1.45	1.40
Tetrachloromethane	0.54	0.52
1,2-Dichloroethane	1.07	1.08
Heptane	−0.12	−0.10
Octane	−0.18	0.08
Nonane	−0.14	−0.01
Cyclohexane	−0.05	−0.05
Benzene	0.79	0.76
Toluene	0.66	0.62
*m*-Xylene	0.55	0.54
Chlorobenzene	0.79	0.75
Bromobenzene	0.75	0.79
Iodobenzene	0.67	0.79
Nitrobenzene	0.88	0.84
Dibutylether	0.14	0.04
Tributylphosphate	0.52	0.72
Methyl isobutylketone	0.57	0.80

For hexane-2,4-dione, log_10_ *P* values into six solvents are known, and yield six corresponding log_10_ *K* values. There are also two further equations in log_10_ *K*_w_ and an equation in GCRI (890), giving a total of 15 equations. From a known refractive index, ***E*** = 0.381 and ***V*** = 0.9854. The 15 equations were solved to yield the descriptors in Table 2 with an *SD* = 0.122 log_10_ units.

There are only four partition coefficients available for heptane-2,4-dione, but these still yield 11 equations (GCRI = 989). A value for ***E*** (0.385) can be obtained from a known refractive index and ***V*** = 1.1263 and the 11 equations solved with *SD* = 0.089 to give the descriptors in Table 2.

In the case of octane-2,4-dione, log_10_ *P* values are known only for partition into heptane and tetrachloromethane, leading to a total of seven equations. ***E*** was estimated as 0.380, ***A*** was taken as zero and ***V*** calculated as 1.2672. The seven equation were solved with SD = 0.031 to give the descriptors in Table 2.

A log_10_ *P* value into tetrachloromethane is all that is available for nonane-2,4-dione. With GCRI = 1188 [13] we have only five equations. ***E*** was estimated as 0.380, ***A*** was taken as zero and ***V*** calculated as 1.4084. This is just enough to obtain the descriptors in Table 2.

Seven log_10_ *P* values are listed for heptane-3,5-dione, and lead to 17 equations (GCRI = 989). An experimental value of the refractive index leads to ***E*** = 0.389, ***V*** is calculated as 1.1263, and the 17 equations can be solved with *SD* = 0.143 to give the descriptors in Table 2.

For octane-3,5-dione only two partition coefficients were available, so that we have seven equations. ***E*** was estimated as 0.380, ***A*** was taken as 0.00 and ***V*** calculated as 1.2672. The equations were solved to give the descriptors in Table 2 with an *SD* of 0.063 log_10_ units.

There is more data for nonane-4,6-dione. We used seven log_10_ *P* values, which translated into 17 equations (GCRI = 1188). A known refractive index gave ***E*** = 0.399 and ***V*** = 1.4081. The set of equations was solved with an SD = 0.167, to give the descriptors in Table 2.

For undecane-5,7-dione we used five partition coefficients, leading to 12 equations. We estimated ***E*** as 0.37, calculated ***V*** as 1.6899 and solved the set of equations to yield the descriptors in Table 2 with an SD value of 0.150 log_10_ units.

As for undecane-5,7-dione we had only five partition coefficients for tridecane-6,8-dione. Taking ***E*** = 0.37 and ***V*** = 1.9717 we solved the set of 12 equations to obtain the descriptors in Table 2 with a rather large SD of 0.178 log_10_ units.

There are also a number of branched chain alkyl derivatives for which partition coefficients are available [12]. For 5,5-dimethylhexane-2,4-dione partition coefficients are known into five solvents, and with GCRI (1004) we have 13 equations. With ***E*** = 0.38 and ***V*** = 1.2672 we solved the set of equations to obtain the descriptors in Table 2; *SD* = 0.128 log_10_ units.

Partition coefficients are available for 2,6-dimethylheptane-3,5- dione and with GCRI = 1060 we had 15 equations. The given experimental value [12] for partition into octanol, log_10_ *P* = 2.22, was well out of line and was omitted. The resulting 13 equations with ***E*** = 0.38 and ***V*** = 1.4081 gave the descriptors in Table 2 with *SD* = 0.162 log_10_ units.

The only partition coefficient that we could find for 2,8-dimethyl-4,6-dione was that of log_10_ *P* = 4.05 for partition into benzene [14], but a value of 1258 was available for GCRI. These yielded only five equations. We estimated ***E*** as 0.37, we know that ***V*** = 1.6899, and in order to solve the equations we also estimated that ***B*** = 0.72. Then solution of the equations gave *SD* = 0.076 and the remaining descriptors as shown in Table 2.

There are two other alkyl derivatives of acac that have been used as complexing agents, 3-methylpentane-2,4-dione and 2,2,6,6-tetramethylheptane-3,5-dione. There is insufficient data on these compounds to yield a set of equations that can be solved to get descriptors, but from the results we have for the other alkyl derivatives, we estimate the descriptors as shown in Table 2.

A number of other derivatives of acetylacetone have been widely used as complexing agents; for several of these compounds, numerous values of log_10_ *P* are known [12]. We start with benzoylacetone (1-phenylbutane-1,3-dione) for which partition into 20 solvents has been studied. A value of 1364 for GCRI is available [13] and so we have no less than 43 equations on the lines of Eqs. 1 and 2. We took ***E*** = 1.00 from addition of fragments and also from calculations [7, 8] and ***V*** = 1.3114. The equations were solved to yield the descriptors in Table 2 with a very small value of SD = 0.086 log_10_ units. The observed and calculated values of log_10_ *P* are in Table 4. For the 20 solvents, *AE* = 0.01 and *SD* = 0.086 log_10_ units

**Table 4:** Calculated and observed values of log_10_ *P* for water–solvent partition of benzoylacetone

Solvent	Calc	Obs
Hexanol	2.46	2.48
Octanol	2.46	2.52
Nonanol	2.37	2.43
Decanol	2.37	2.38
Dichloromethane	3.44	3.48
Trichloromethane	3.52	3.50
Tetrachloromethane	2.79	2.77
1,2-Dichloroethane	3.24	3.23
Heptane	1.99	2.03
Octane	1.98	2.00
Nonane	1.97	1.97
Decane	1.95	2.00
Cyclohexane	2.21	2.11
Benzene	3.10	3.05
Toluene	2.96	2.91
m-Xylene	2.83	2.81
Chlorobenzene	3.13	3.13
Bromobenzene	3.06	3.36
Nitrobenzene	3.25	3.19
Dibutylether	2.50	2.36

Partitions into 11 solvents are known for 1,1,1-trifluorobenzoylacetone. The value of log_10_ *P* into trichloromethane was quite out of line (obs. 2.73, calc. 3.28) and if this is left out we have 23 equations (GCRI = 1198). We estimated ***E*** = 0.69 from values for pentane-2,4-dione, 1,1,1-trifluoropentane-2,4-dione and benzoylacetone, and calculated ***V*** = 1.3645. The set of equations were solved to give the descriptors in Table 2 with SD = 0.126 log_10_ units.

For thenoylacetone (1-(2-thienyl)butane-1,3-dione) we have log_10_ *P* values into hexane and benzene. Together with a value of 1385 for GCRI these gave seven equations. We estimated ***E*** = 1.10 by addition of fragments, calculated ***V*** as 1.2361 and solved the equations to give the descriptors in Table 2 with SD = 0.039 log_10_ units.

There are a large number of log_10_ *P* values available for trifluorothenoylacetone **(**4,4,4-trifluoro-1-(2-thiényl)butane-1,3-dione). These include values for partition into numerous esters for which we have no coefficients in Eqs. 1 and 2. For partition into ethyl acetate and butyl acetate, however, the observed values of log_10_ *P* are so far away from our calculated values that we suggest all the given log_10_ *P* values into esters be used with caution. We were left with 12 values of log_10_ *P*, together with a value of 1199 for GCRI, leading to 27 equations. A calculated refractive index [10] leads to ***E*** = 0.524, close to a calculated value for ***E*** of 0.53 [8]. We used the latter value and our calculated value of ***V*** = 1.2892, and solved the 27 equations to give the descriptors in Table 2 with an SD of 0.101 log_10_ units.

There are also log_10_ *P* values for 2-furoyltrifluoroacetone and pivaloyltrifluoroacetone, but we could not obtain any reasonable set of descriptors for these two compounds.

Finally we deal with trifluoroacetylacetone (1,1,1-trifluoropentane-2,4-dione) and hexafluoroacetylacetone (1,1,1,3,3,3-hexafluoropentane-2,4-dione). For trifluoroacetyl-acetone we have log_10_ *P* values into 15 solvents. The value of log_10_ *P* into trichloromethane was considerably out of line (calc. 0.94, obs. 0.33) and was left out. With GCRI = 624 this leaves 31 equations to solve. An experimental refractive index of 1.3890 [13] leads to ***E*** = 0.106 and with ***V*** = 0.8976 we obtained the descriptors in Table 2 with an *SD* of 0.125 log_10_ units. The calculated and observed values of log_10_ *P* are in Table 5, and yield *AE* = 0.011 and *SD* = 0.123 log_10_ units (omitting trichloromethane). It is noteworthy that the ***A***-descriptor is not zero, but with a set of 31 equations, we can be reasonably confident about this descriptor.

**Table 5:** Calculated and observed values of log_10_ *P* for water–solvent partition of trifluoroacetylacetone

Solvent	Calc	Obs
Pentanol	0.72	0.52
Hexanol	0.67	0.62
Nonanol	0.49	0.59
Dichloromethane	0.70	0.40
Trichloromethane^a^	0.94	0.33
Tetrachloromethane	−0.01	0.02
Hexane	−0.52	−0.51
Heptane	−0.55	−0.57
Octane	−0.63	−0.62
Dodecane	−0.75	−0.76
Benzene	0.13	0.15
Toluene	0.04	0.13
Ethylbenzene	−0.10	−0.18
Nitrobenzene	0.10	0.30
Dibutylether	0.04	0.08

^a^Not used in the calculations of descriptors

The position with hexafluoroacetylacetone is not straightforward. We have four values [15] of log_10_ *P* into trichloromethane (−1.75), tetrachloromethane (−1.92), hexane (−2.04) and benzene (−1.91), and also a value of GCRI (459), leading to eleven equations. The solution of this set of simultaneous equations yields completely unreasonable values for the descriptors. Stokely [16] has shown that hexafluoroacetylacetone decomposes in water. He measured a value for log_10_ *P* into benzene of −0.42 (in contrast to the value of −1.91 [15]), and found that the partition coefficient decreased with time. We can obtain a value of −0.217 for ***E*** from the refractive index and we can calculate ***V*** = 0.9507, but there is still not enough data to obtain a full set of descriptors. We can deduce that ***B*** = 0.80 and ***L*** = 2.340 by comparison to other compounds in Table 2, and from Absolv calculations [7]. Then with ***S*** = 0.07 and ***A*** = 0.32 we can reproduce Stokely’s [16] value of −0.42 for log_10_ *P* into benzene, and the associated values of log_10_ *K* into benzene and log_10_ *K*, with the descriptors in Table 2. However, we caution that these results must be regarded as provisional only.

## Discussion

We have managed to obtain descriptors for acetylacetone and 21 of its derivatives, as shown in Table 2. These can be combined with the equation coefficients in Table 1 to yield estimates of partition coefficients from water and the gas phase into all the listed solvents, and (hypothetical) partition coefficients into a large number of dry solvents for which we have also determined equation coefficients [17–19]. In addition we have determined equation coefficients for partition into water–ethanol [20, 21] and water–methanol mixtures [22], and so values of log_10_ *P* and log_10_ *K* into these solvent mixtures can also be estimated. In addition to the usual organic solvents, we have also studied ionic liquids [23], and partitions into these solvents can be estimated for the various acetylacetonates. Partitions or permeations in biological systems [24–26] can also be estimated from the descriptors listed in Table 2.

Inspection of the descriptors themselves shows that all the acetylacetonates are quite polar, with substantial values of the ***S***-descriptor, and, as expected from the presence of the two carbonyl groups, are quite strong hydrogen bond bases, with ***B***-values almost double those for simple aliphatic ketones which have ***B*** **-**values around 0.45 [7, 8]. Perhaps unexpectedly, the alkylsubstituted acetylacetonates all have zero hydrogen bond acidity, as do some of the trifluoroderivatives. Only with trifluoroacetylacetonate, and with hexafluoroacetylacetonate are significant values of the ***A***-descriptor found.

The ***L***-values form a very regular series, and can be taken to show the internal consistency of our set of descriptors. For the acetylacetonates with linear alkyl substituents we find Eq. 5, where CN is the number of carbon atoms.

5 $$ \varvec{L} = \, 0. 7 1 3 2 { } + \, 0. 50 6 9 {\text{ CN}} $$

$$ N = { 1}0,SD = \, 0.0 5 9,R^{2} = \, 0. 9 9 8,F = { 3734}.0 $$

$$ {{PRESS }} = \, 0.0 6 2 3 5,Q^{2} = \, 0. 9 9 5,PSD = \, 0.0 8 9 $$

The branched chain substituents behave remarkably similarly to the linear chain substituents, and for all the alkyl substituted acetylacetonates we find Eq. 6.

6 $$ \varvec{L} = \, 0. 7 5 1 6 { } + \, 0. 50 1 5 {\text{ CN}} $$

$$ N = { 15},SD = \, 0.0 5 3,R^{2} = \, 0. 9 9 8,F = { 6169}. 3 $$

$$ PRESS = \, 0.0 6 1 6 4 40,Q^{2} = \, 0. 9 9 7,PSD = \, 0.0 6 9 $$

**Fig. 1:**
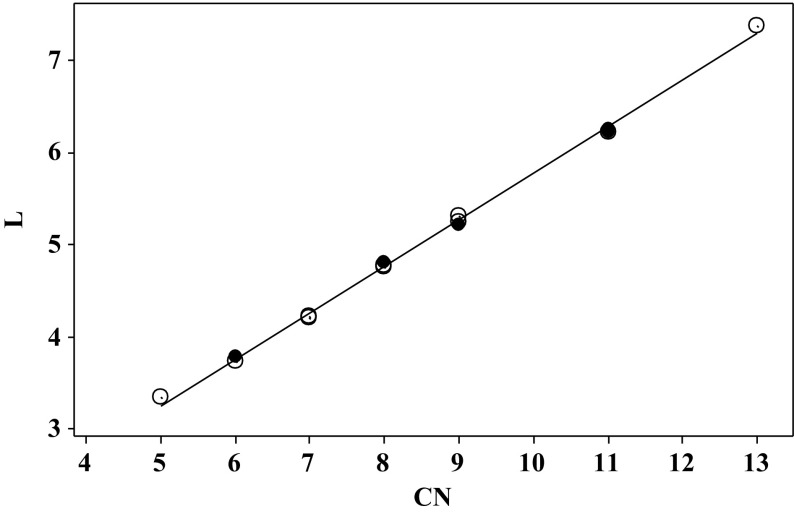
Plot of the descriptor ***L*** against the number of carbon atoms, C**N**, in alkyl prentane-2,4-diones, *open circle* linear alkylpentane-2,4-diones, *filled circle* branched chain alkylpentane-2,4-diones

The plots of ***L*** against CN are excellent, as shown in Fig. 1. Equation 5 or especially Eq. 6 could be used to estimate an ***L***-value for any alkylsubstituted acetylacetonate.

Once we have descriptors for the acetylacetonates, we can then deduce the corresponding water–octanol partition coefficients, as log_10_ *P*. These partition coefficients are of considerable interest, as they are often used as a measure of hydrophobicity of solutes, and they are the most commonly estimated of all water–solvent partition coefficients. We can compare our own calculated values with those calculated through four very common methods, the ClogP program of Leo [12], the EPI Suite TM [27], the ACD program in ChemSketch [10] and the ACD program that is part of the Absolv ADME Suite [7]. In addition, we can compare all the calculated values with the (few) observed values. Details are in Table 6. There are eight compounds for which observed values are available, and a comparison of these with the various calculated values, in terms of the average error and standard deviation, is in Table 7.

**Table 6:** Observed and calculated values of log_10_ *P* for acetonylacetonates

		Calc			acd	PHA
Compound	Obs	This work	[12]	[27]	[10]	[7]
Pentane-2,4-dione	0.40	0.55	−0.46	0.05	0.34	0.58
Hexane-2,4-dione		0.99	0.07	0.54	0.87	0.91
Heptane-2,4-dione		1.62	0.60	1.03	1.40	1.28
Octane-2,4-dione		2.12	1.13	1.53	1.93	1.76
Nonane-2,4-dione		2.59	1.65	2.02	2.46	2.39
Heptane-3,5-dione		1.50	0.60	1.02	1.40	1.28
Octane-3,5-dione		2.09	1.13	1.53	1.93	1.76
Nonane-4,6-dione	2.23	2.59	1.65	2.02	2.46	2.24
Undecane-5,7-dione	3.56	3.59	2.71	3.00	2.99	3.53
Tridecane-6,8-dione	4.88	4.71	3.77	3.98	4.59	4.39
5,5-Dimethylhexane-4,6-dione	1.67	1.75	1.00	1.41	1.57	1.67
2,6-Dimethylheptane-3,5-dione	2.22	2.66	1.65	1.87	2.10	2.09
2,8-Dimethylnonane-4,6-dione		3.44	2.45	2.85	3.16	2.92
3-Methyl-2,4-pentanedione		1.00	0.07	0.47	0.69	0.92
2,2,6,6-Tetramethylheptane-3,5-dione		3.42	2.45	2.78	2.80	2.91
Benzoylacetone	2.52	2.46	1.09	0.91	2.52	1.85
1,1,1-Trifluorobenzoylacetone		2.29	1.65	1.02	4.17	2.54
Thenoylacetone		1.98	0.86	0.43	2.11	1.29
1,1,1-Trifluorothenoylacetone	1.46	1.47	1.42	0.84	3.76	1.99
Trifluoroacetylacetone		0.59	0.10	0.47	2.09	1.32
Hexafluoroacetylacetone		0.76	0.66	0.88	3.84	1.69

**Table 7:** Comparison of observed and calculated values of log_10_ *P* for acetonylacetonates

	This work	[12]	[27]	[10]	[7]
AE^a^	−0.10	0.76	0.60	−0.17	0.08
SD	0.23	0.91	0.80	0.91	0.38

^a^Obs – Calc

Inspection of Tables 6 and 7 suggests that where our descriptors are available, they yield estimates of water–octanol partition coefficients, as log_10_ *P*, that are at least as good as those from standard calculation methods [7, 10, 12, 27]. In addition, use of our descriptors has the advantage that water–solvent partition coefficients can be estimated for a very large number of organic solvents. The deviations in observed and calculated values of log_10_ *P* for the eight acetonylacetonates are quite similar thus indicating that the errors in the descriptors for the various acetonylacetonates are also quite close.

## Conclusions

We have been able to obtain Abraham or Absolv descriptors for pentane-2,4-dione and 21 of its derivatives. These descriptors encode important chemical properties, and show that pentane-2,4-dione itself has no hydrogen bond acidity, but that the trifluoro- and hexafluoro-derivatives have substantial hydrogen bond acidity. The descriptors for the 22 compounds enable partition coefficients to be estimated for partition from water to a very large number of organic solvents. In the case of water–octanol partition coefficients we show that estimations through our descriptors are at least as good as the best calculations through well-known calculational programs.
